# Will Happiness-Trainings Make Us Happier? A Research Synthesis Using an Online Findings-Archive

**DOI:** 10.3389/fpsyg.2020.01953

**Published:** 2020-11-17

**Authors:** Ad Bergsma, Ivonne Buijt, Ruut Veenhoven

**Affiliations:** ^1^Erasmus Happiness Economics Research Organization, Erasmus University Rotterdam, Rotterdam, Netherlands; ^2^Optentia Research Program, North-West University, Vanderbijlpark, South Africa

**Keywords:** affect balance, happiness, happiness training, happiness awareness, life skills, life satisfaction, positive psychology interventions, research synthesis

## Abstract

Most people want to be happy and many look out for opportunities to achieve a more satisfying life. Following a happiness training is an option, but the effectiveness of such training is being questioned. In this research synthesis we assessed: (1) whether happiness training techniques add to the happiness of their users, (2) how much happiness training techniques add to happiness, (3) how long the effect of happiness training lasts, (4) what kinds of training techniques work best, and (5) what types of groups of people profit from taking happiness training. We took stock of the available research and found 61 reports of effect studies on training techniques, which together yielded 179 findings. These findings are available in an online “findings archive,” the World Database of Happiness. Using links to this source allows us to condense information in tabular overviews, while providing the reader with access to much detail. Happiness training techniques seem to do what they are designed to do: 96% of the studies showed a gain in happiness post intervention and at follow-up, about half of the positive results were statistically significant. Studies with cross-sectional designs and studies that used control groups showed more mixed results. The average effect of happiness training was approximately 5% of the scale range. We conclude that taking a form of happiness training is advisable for individuals looking for a more satisfying life. Since happier workers tend to be more productive, organizations would be wise to provide such training techniques for their workforce.

## Introduction

### Call for Greater Happiness

Most people want to be happy, and many of them look for opportunities to achieve a more satisfying life (Diener et al., 1998). This pursuit seems to be universal, but it is particularly pronounced in modern societies (Veenhoven, 2015). One reason for the heightened interest in happiness is the greater awareness that we have considerable control over our happiness. Happiness is no longer considered a matter of fate (Nes and Røysamb, 2017), but rather a condition that can actively be pursued, developed, and sustained (Sezer and Can, 2019) and that is a personal responsibility (Elliott and Lemert, 2009). Sheldon and Lyubomirsky (2019) argued that 40% of one’s level of happiness is a function of purposeful and intentional action, although that may be an overestimation (Brown and Rohrer, 2019). Another reason for the call for greater happiness is the rising evidence of the positive effects of happiness on other areas of life such as health (Veenhoven, 2008) and civil behavior (Guven, 2008). Employers are keen to raise happiness in their workforce, particularly in view of the evidence that life satisfaction fosters productivity more than job satisfaction (Gaucher and Veenhoven, 2020; Bergsma and Veenhoven, 2020).

#### Happiness Education

The call for greater happiness is met in two ways: by improving external living conditions and by strengthening life skills that enable people to live in the upper range of their happiness potentials (Sheldon and Lyubomirsky, 2019). A new field of research and practice centers around structured training and educational initiatives designed to strengthen individuals’ life skills. This field is aptly labeled “happiness education” and is comparable to, and often intertwined with, existing “health education.” Happiness education can be found in a growing number of advisory books, on self-help websites, and at the mounting supply of (online) courses on happiness (Bergsma, 2008; Parks et al., 2013). Alongside such education, a practice of happiness coaching has developed (Grant and Spence, 2010; Freire, 2013). Professional life coaches offer advice on how to live a more rewarding life, and they have gained a greater share of the work of psychologists and social workers (Tarragona, 2015). These developments are inspired by the scientific fields of “positive psychology” and “positive education,” which came into existence around the year 2000 and added scientific rigor to practices in the expanding training sector (Boniwell, 2012). Positive psychology interventions (PPIs) have been developed with the aim of strengthening people. These interventions typically consist of a combination of teaching and exercises. The common aims of such training techniques are to get individuals to see and seek meaning in their work and lives, to know who they are, and to foster positive feelings and self-reliance (Sin and Lyubomirsky, 2009).

### Happiness Training Techniques

One kind of PPI focuses on increasing satisfaction with one’s life. This kind is commonly presented as “happiness training” (Fordyce, 1977). These training techniques help an individual to gain insight into the sources of their happiness and to learn skills that are functional for living a happy life (Feicht et al., 2013). The focus of these training techniques is not on a specific life domain, such as work or marriage, but on one’s life as a whole (Bergsma and Veenhoven, 2020). An advanced Google search on “happiness training” yielded 69,800 hits in December 2019. Some examples are the “Happiness Training Plan” (College of Well-being, n.d.), the Buddhist-inspired online course “A Life of Happiness and Fulfillment” (Indian School for Business, n.d.), and the Action for Happiness Course (Action for happiness, n.d.).

### Doubts About the Effectiveness of Happiness Trainings

The majority of happiness training techniques focus on individuals. Happiness training techniques applicable to organizational contexts are still underdeveloped and not often utilized (Nielsen et al., 2017) since organizations focus on work-related skills and engagement rather than on wider life skills (Ivandic et al., 2017; Donaldson et al., 2019a, b; Roll et al., 2019). One of the reasons for this could be existing doubts about the effectiveness of happiness training interventions (Donaldson et al., 2019b). These doubts are rooted in theories of happiness and in reservations about PPIs in general and about happiness training in particular.

#### Qualms About the Possibility of Greater Happiness

There are doubts that the level of individual happiness can be raised because, among other concerns, happiness is believed to depend on social comparison. In this view, people are happier if they think they are better off than others, making happiness a zero-sum game (Brickman and Campbell, 1971). Others claim that happiness is part of a fixed genetic disposition and therefore determined by personality traits that remain constant (e.g., Omerod, 2012). A third reason is that the conscious pursuit of happiness may be self-defeating because higher expectations of happiness will lead to frustration if not realized (e.g., Ford and Mauss, 2014), which implies that the use of a happiness training technique will decrease one’s happiness. A fourth reason is that the pursuit of happiness stimulates people in individualistic societies to focus on individual goals, whereas more socially engaged ways to seek happiness are deemed more effective (Ford et al., 2015). Looking for happiness may even increase loneliness (Mauss et al., 2012), and valuing happiness may give rise to depression (Ford et al., 2014). Chasing happiness may also be self-defeating if people seek more positive effects directly, while, in contrast, aiming to fulfill basic psychological needs of relatedness, autonomy, and competence may yield better results (Sheldon and Lyubomirsky, 2019). Although most of these doubts have been discarded in the scientific literature (Veenhoven, 2010), they still live in public opinion. The dark sides of the pursuit of happiness, as well as the caveats and limitations, have a higher attentive value for the media than the stories with a happy ending (Soroka and McAdams, 2015).

#### Limited Effects of Positive Psychological Interventions (PPIs) in General

**FIGURE 1:**
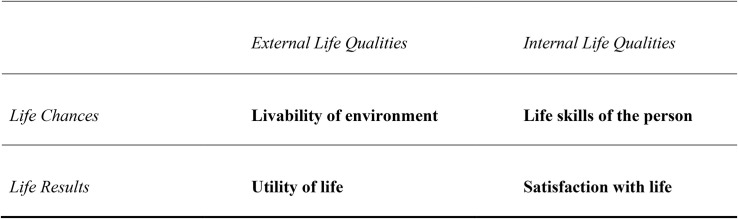
Four Qualities of Life. Source: Veenhoven (2000, 2019c).

Three major meta-analyses on the effectiveness of PPIs have not yielded impressive effects. Sin and Lyubomirsky (2009) reported a modest effect (mean r = +0.29, median r = +0.24) on “well-being.” These numbers are difficult to interpret because the studies covered different notions of well-being, most of which belong in the life-ability quadrant of Figure 1 (see below). Bolier et al. (2013) report a smaller effect (d = +0.34) on subjective well-being that partly waned at follow-up (d = +0.22) and after the removal of outliers (d = +0.17). The authors were not very specific about the subjective well-being measures they included. Multi-component PPIs have a small to moderate effect on subjective well-being (Hedges’ g = +0.34), but again, the authors were not very specific on the subjective well-being measures they included. The removal of outliers or low-quality studies lowered the effect on well-being (g = + 0.24 without outliers, g = +0.26 for high-quality studies) (Hendriks et al., 2019). The modest effects of the meta-analyses we described may be too high because negative findings tend to be underreported in scientific literature. A recent re-analysis of the studies included in the first two meta-analyses mentioned above used an improved correction for small sample sizes and found an effect of 0.1 of PPIs on well-being (White et al., 2019).

#### Reservations About Happiness Training Techniques in Particular

In a recent Delphi study by Buettner et al. (2020), 14 leading scientists rated the effectiveness of “Ways to Greater Happiness” on a five-step scale. Their effectiveness rating for “Develop skills for greater happiness, using self-help or professional coaching” was 3.1, while their average rating for methods such as “Invest in friends and family” and “Get physical exercise” was about 4.

The general public seems to have a mixed attitude toward happiness advice and training. There is much interest but also a lot of skepticism and grumbling about the “tyranny of positivity” (Held, 2002, 2018). One of the reasons may be that the term “happiness” is used to promote the particular trendy practices of the moment, such as meditation and veganism. This is part of the wider problem of the term “happiness” being increasingly used in sales communication as a “feel-good” term (e.g., Coca-Cola with its “Open Happiness” slogan). A shared definition of happiness is lacking, and this is another reason to question the message of happiness coaches and trainers.

### Research Questions

Are these doubts about the effectiveness of happiness training techniques justified? In this study we seek to answer the following questions:

• Do happiness training techniques add to happiness?
  ○ If so, how strong is the effect?
  ○ If so, how long-lasting is the effect?
• What kind(s) of training techniques work best?
  ○ What nature of training techniques works best?
  ○ What modes of training techniques work best?
• What types of people profit most from joining a happiness training course?

### Concept of Happiness

In answering these questions, we focus on happiness in the sense of “life satisfaction,” which we will define in detail below. To our knowledge, the research literature on this subject has not been reviewed with that specific definition in mind.

#### Meanings of the Word

In a broad sense, the word happiness is used to denote a “good life” and used as a synonym for “quality of life” or “well-being.” This meaning prevails in moral philosophy where it serves as a starting point for speculations about what qualities make the best life, such as the importance of “wisdom” (McMahon, 2018). In contemporary social sciences the term is increasingly used for one particular quality of life, that is, how satisfying one’s life is. Since this is a measurable phenomenon, its determinants can be identified inductively using empirical research (Diener et al., 2015).

#### Definition of Happiness

Happiness is defined as the degree to which individuals judge the overall quality of their life as a whole favorably (Veenhoven, 1984). This definition fits the utilitarian tradition and is most closely associated with Bentham’s (1789) view of happiness, which is described as “the sum of pleasures and pains” (Veenhoven, 2009). This concept is central in the World Database of Happiness, which we draw from for this research synthesis.

#### Other Notions of Quality of Life and Satisfaction

We realize that some readers will associate “happiness” with other notions of well-being, in particular readers with a background in positive psychology where the term “eudaimonic well-being” is currently used for positive mental health (Delle Fave et al., 2011). Therefore, we are expanding on this difference using Veenhoven (2000) classification of four qualities of life. This classification is based on two distinctions: vertical and horizontal. Vertically, there is a difference between opportunities and actual outcomes of life. This distinction is important because people can fail to use the life chances offered to them. The horizontal distinction refers to external qualities of the environment and internal qualities of the individual. Together, these two dichotomies produce four qualities of life, all of which have been denoted by the word “happiness.”

In Figure 1, our concept of happiness is positioned in the right-bottom quadrant, as an inner outcome of life. Positive mental health (eudaimonic happiness) belongs in the top-right quadrant of Figure 1, that is, as a precondition for happiness. We only include measures of happiness that belong to the right-bottom quadrant. Our conceptual focus is sharper than that of earlier meta-analyses of positive psychological interventions, which included measures of well-being that also cover other quadrants of Figure 1. As such, our results are easier to interpret.

#### Components of Happiness

The overall evaluation of life draws on two sources of information: (1) how well we feel most of the time, and (2) to what extent we perceive that we are getting from life what we want from it. We refer to these sub-assessments as “components” of happiness, respectively called “hedonic level of affect” and “contentment” (Veenhoven, 1984). Diener et al. (1999) make a similar distinction between affective and cognitive appraisals of life, but do not conceptualize an overall evaluation in which these appraisals are merged. In this research synthesis we include all three variants, overall happiness and its two components.

## Materials and Methods

**FIGURE 2:**
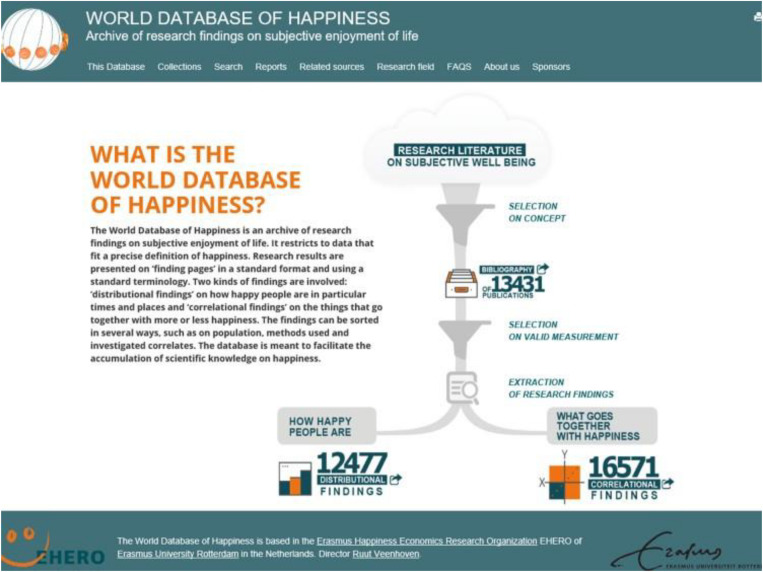
Start page of the World Database of Happiness, showing the structure of this findings archive.

We seek answers to the research questions mentioned above in section “Research Questions” by taking stock of the available research findings. For this purpose, we draw on the World Database of Happiness (Veenhoven, 2019g, 2020). This is a “findings archive” that contains some 20,000 abstracts of observed correlations with happiness, presented on electronic “finding pages” in a standard format and terminology. The structure of this “finding archive” is presented on Figure 2. The finding pages are sorted by subject, and one of the subject categories is “happiness training,” which contained 179 findings in December 2019. The World Database of Happiness restricts to findings obtained with measures of happiness that fit the above-mentioned definition of happiness, the selection of which is explained below. The use of this findings archive implies another way of gathering the available research findings than is usual in review studies and provides new ways for presenting the data. The technique is described in detail in Veenhoven (2020). We call it a “support system for research synthesis.” In section “Advantages and Disadvantages of Using an Online Finding Archive,” we discuss the advantages and disadvantages of this method. In section “Differences With Meta-Analysis,” the differences of this research synthesis with meta-analysis are discussed.

### Search Strategy

The World Database of Happiness, and hence this research synthesis, restricts to research findings on happiness as defined above. Publications on that matter have been gathered on a continuous basis since 1980 – at the outset, mainly with searches in abstract systems such as the Web of Science, and today by also tracking references in publications and using announcement services, such as New Economic Papers on Happiness (for detail Veenhoven, 2019b). Selected publications are entered in the Bibliography of Happiness and classified by main subject addressed, one of which is “happiness training” (Veenhoven, 2019a). We updated this latter collection with an additional literature search in Google Scholar and by tracking references in reviews of research on effects of PPIs.

### Eligibility Criteria

#### Measurement of Happiness

Since happiness is defined as something we subjectively experience, it can be measured using questioning. Not all questions used for measuring happiness studies fit the above definition of happiness. The World Database of Happiness, and hence this research synthesis, restricts to findings yielded with measures of happiness that have passed a face-validity test and are listed in the “Collection Measures of Happiness” in the World Database of Happiness (Veenhoven, 2019e).

#### Rejected Happiness Scales

Several multiple-item happiness scales failed this test because they include questions on other kinds of well-being than happiness as defined above. This is the case for two “happiness scales,” that are often used in studies on the effects of happiness training techniques, the Satisfaction With Life Scale (SWLS) by Diener et al. (1985) and the Subjective Happiness Scale (SHS) by Lyubomirsky and Lepper (1999). Both are multiple-item questionnaires that contain one or more questions that does not fit our definition of happiness. In the case of the five-item SWLS, the statement “If I could live my life over again, I would change nothing.” does not fit. Logically, one can be satisfied with life, but still be open for something else. The item is particularly inapt for measuring the effects of happiness training techniques since users of such techniques typically seek change in their lives. In the case of the four-item SHS, the problem is in the statement “Compared to other people, I consider myself less happy/more happy.” Logically, one can think one might be happier than other people, but still be unhappy. Practically, we are often poorly informed of how happy “other” people are. In our view, this lack of substantive face validity cannot be offset by mathematical “tests” for concurrent validity or construct validity. Consequently, we excluded data yielded with these measures, sacrificing a number of findings to maintain a clear meaning of the remaining findings.

#### Valid Measures Included

Three types of accepted happiness measures are included in this research synthesis: (1) measures of overall “life satisfaction,” (2) measures of “hedonic level of affect,” that is, the affect balance scores and answers to the question about how happy one feels, and (3) mixed measures that combine questions about life satisfaction and affect level. The full text of these measures is available behind links in Table 1. Measures of “contentment” have not been used in any of the studies included in this research synthesis.

**TABLE 1:** Studies included in this research synthesis.

Target group: Country and year	N	Happiness training		Type of happiness measure^a^	Source^b^
				
		Named in WDH	Named by author		
**Patients**					
Depressed adults, UK, 2009	55	Goal setting training	Self-help, positive goal-focused intervention	Affect balance Life-satisfaction	Coote & Macleod, 2012
Medical patients with neuromuscular disease, USA, 1998	65	Gratitude training	Happiness training; gratitude exercises	Affect balance Feel happy	Emmons & McCullough, 2003
Poor-health participants in a course of mind-body therapies, Sweden, 2000–2001	152	Meditation, mindfulness	Health self-management course	Affect balance	Fernros et al. 2008
Home-bound elderly, USA, 1982	51	Life-review exercise	Life-review program	Affect balance	Haight, 1988
Distressed adults, Netherlands, 2005	57	Meditation, mindfulness	Mindfulness based stress reduction	Affect balance	Nyklícek & Kuijpers, 2008
Patients on a waiting list for psychological treatment, Australia, 2012	48	1) Gratitude training 2) Kindness training	Gratitude and kindness interventions	Feel happy Affect balance	Kerr et al. 2015
**Students**					
Psychology students, USA, 2005	180	Life-review exercise	Positive reminiscence training	Feel happy	Bryant et al. 2005
Students, USA and South Korea, 2007	218	Kindness training	Performing acts of kindness with or without and autonomy support	Affect balance	Della Porta, 2013
Students, USA, 1998	166	Gratitude training	Count blessings, thinking about five hassles, social comparison	Affect balance	Emmons & McCullough, 2003
Students in well-being course, USA, 2003	192	(1) Gratitude training (2) Life-review exercise	Count blessings		
University students, Turkey, 2015	72	Practice retrospective sources of happiness	Increasing activities and engagement	Affect balance	Eryilmaz, 2015
Students, USA, 1972	202	Training for multiple mental skills	Happiness training	Feel happy	Fordyce, 1977
University students, USA, 1980	57	Training for multiple mental skills	Program to increase personal happiness, aiming to change 14 fundamental behaviors	Feel happy	Fordyce, 1983
	71				
	98				
	57				
	69	Happiness education			
Students, Italy, 2001	92	Training for multiple mental skills	Subjective well-being training course	Feel happy	Goldwurm et al. 2003
Psychotherapy students, Italy, 2004?	80	Cognitive reframing	Subjective wellbeing training	Feel happy	Goldwurm et al. 2006
Student participants in a savoring exercise,USA, 2011	193	Savoring training	Recalling positive events in the past week	Affect balance	Hurley & Kwon, 2012
Students,United Arab Emirates, 2015	267	Training for multiple mental skills	PPI program	Affect balance	Lambert et al. 2019
Students participating in happiness training,USA, 2014	139	Lifestyle awareness training	Time scarcity training	Affect balance	Layous, et al. 2018
Psychology students,New Zealand, 1978	48	(1) Self-awareness training (2) Positive thinking training	Cognitive retraining	Affect balance	Lichter et al. 1980
Psychology students, Germany, 2014	349	Mood tracking	Use of Happiness Analyzer	Affect balance Feel happy Life satisfaction	Ludwigs et al. 2018
Students,USA, 2006	96	Life-review exercise	Writing, talking, and thinking about life’s triumphs and defeats	Affect balance	Lyubomirsky et al. 2006
Students and people from local education center, UK, 2008	64	Goal setting training	Goal setting and planning training	Affect balance	MacLeod et al. 2008
Psychology students, Spain, 2010	105	Gratitude training	Gratitude writing intervention	Feel happy	Martinez-Marti 2010
University students, South Korea, 2009	50	(1) Goal setting training (2) Practice one’s values	Well-being training	Affect balance	Nelson et al. 2014
Psychology students, USA, 2009	62	Practice one’s values	Self-affirmation	Affect balance	Nelson et al, 2014
Students, USA, 2004	360	Training for multiple mental skills	Positive psychotherapy training	Life satisfaction	Parks, 2004
Student participants in a happiness training, USA, 2007–2008	267	Training for multiple mental skills	Positive psychotherapy training	Life satisfaction	Parks 2009
Psychology students, USA, 2000	90	Goal-setting training	Goal training intervention, growth training	Affect balance	Sheldon et al. 2002
**School children**					
Middle school students, Netherlands, 2009	631	Training for multiple mental skills	Lessons in happiness	Feel happy	Boerefijn & Bergsma 2011
School children aged 9–11, UK, 2014	606	Positive thinking training	Positive psychology intervention	Feel happy	Carter, 2016
Students aged 12–17, USA, 2006	221	Gratitude training	Gratitude increasing intervention	Life satisfaction	Froh et al. 2008
Pupils of a parochial school aged 8–19, USA	89	Gratitude training	Gratitude intervention	Affect balance	Froh et al. 2009
Children aged 9–12, Netherlands, 2012	183	Training for multiple mental skills	Happiness lessons	Feel happy	Leeuw, 2012
School children aged 10–12, USA, 2008	55	Positive thinking training	Wellness program	Affect balance	Suldo et al 2014
**Self-selected users of happiness trainings**					
Meditation trainees, Oman, 2001	45	Meditation, mindfulness	Meditation course	Feel happy	AlHusani, 2001
Users of the “Happiness Indicator” self-help website	5411	Comparison with the happiness of similar people	Happiness comparer	Feel happy	Bakker et al. 2020
		Mood awareness training	Happiness diary		
Participants in a mindfulness meditation course, USA, 2008	69	Meditation, mindfulness	Mindfulness training	Life-satisfaction combined with Affect balance	Brown et al. 2009
Long-term meditators, Netherlands, 2009	20	Meditation, mindfulness	Long-term meditation	Affect balance	Choi, 2011
Participants in a 9-day meditation retreat, Netherlands, 2009	26	Meditation, mindfulness	9-day vipassana meditation retreat	Affect balance Feel happy combined with life-satisfaction	
Healthy adult volunteers, USA, 2005	73	Meditation, mindfulness	Sacred moments intervention	Affect balance	Goldstein, 2007
Users of an online training, 35 nations, 2012	270	Training for multiple mental skills	Psycho-education course on positive psychology	Affect balance	Haeck, et al. 2016
Participants in a gratitude training, Poland, 2016	58	Gratitude training	Gratitude exercise	Affect balance	Krejtz et al. 2016
Adult volunteers, New Zealand, 1978	33	Multiple kinds of happiness trainings	Course on happiness and positive mental health	Life satisfaction Feel happy	Lichter et al. 1980
Participants in a savoring training, Spain, 2017	150	Savoring training	Appreciation of beauty	Affect balance	Martinez-Marti et al. 2018
Users of a mood-tracking website, USA, 2010	5952	Mood awareness training	Frequent use of mood tracker	Affect balance	Moodscope, 2010
Participants in a happiness training, USA, 2004	37	Training for multiple mental skills	Positive psychotherapy training	Life satisfaction	Parks, 2004
Users of online self-help program, USA, 2007	327	Training for multiple mental skills	“Live happy” online self-help program	Affect balance	Parks et al 2012
Participants in a 4-week psychological training, Canada, 2011	65	(1) Gratitude training (2) Life-review exercise	Gratitude inducing exercises	Affect balance	Rash et al. 2011
Participants in a happiness training, USA, 2011	79	(1) Novelty training (2) Goal setting training	Hedonic adaptation prevention	Affect balance	Sheldon et al, 2013
Participants in a happiness training, USA, 2004	113	List and practice perceived ways to happiness	Change your actions not your circumstances	Affect balance	Sheldon & Lyubomirsky, 2009
Participants in a yoga course, Australia, 2007	88	Meditation, mindfulness	Laughter yoga	Life satisfaction	Weinberg et al, 2014
**Miscellaneous groups**					
School teachers, Hong Kong, 2007	89	Gratitude training	Gratitude intervention program	Affect balance	Chan 2010
Young adults, Spain, 201?	78	Lifestyle awareness training	Best possible self-Intervention	Affect balance	Enrique et al. 2018
Self-selected employees, Germany, 2012	147	Training for multiple mental skills	7-week web-based happiness training	Life satisfaction Feel happy	Feicht et al. 2013
Managers participating in personality development course, Germany, 2000	99	Empowerment training	Goal-setting training	Affect balance	Kehr, 2003
Self-selected older adults, UK, 2014	88	Savoring training	Gratitude intervention	Affect balance	Killen & MacasKill, 2015
Employee well-being trainees, Australia, 2009	31	Training for multiple mental skills	Well-being training program	Affect balance	Page & Vella-Brodrick, 2013

**^*a*^Click linked text to find the specific happiness measure used in the*World Database of Happiness. *^*b*^Click linked text to find the excerpt of the study in the*World Database of Happiness.*

#### Happiness Training

Happiness training techniques are a kind of PPI. In practice, it is often difficult to see what particular training techniques precisely aim to improve. The first problem is in the naming of interventions; the use of the term “happiness” in the appellation does not always mean that happiness as defined above is targeted (e.g., this is not the case for the Happiness Course (The happiness course, n.d.), which is about strengthening religious communities). A second problem is that training techniques presented as a “happiness training” often aim to meet multiple goals, of which happiness is only one, and where “happiness” is seldom clearly defined. A third problem is that there are training techniques that focus on happiness, but do not use the word “happiness” in their name, as with, for example, the “Growth Training” developed by Sheldon et al. (2002).

We dealt with these problems in the following ways. First, we ignored the specific name used for a training technique. Instead, we looked at the instructions and materials to see what was actually trained. Another indication for the aim of the training was the outcome measures used in studies on its effects. This worked in most of the cases but was not always clear. This research synthesis may therefore not be complete, but it does offer a pragmatic grasp of studies.

### Studies Selected

At the end of 2019, the World Database of Happiness included 61 studies in which the effects of happiness training on happiness had been assessed using a valid measure of happiness. These 61 studies were reported in 54 publications, which had been published between 1972 and 2019. Together, these 61 studies report 179 “findings,” since several studies report more than one result. A list of these studies is presented in Table 1. The links in Table 1 lead to the specific happiness measures used and to the excerpts of the studies in the World Database of Happiness.

#### People Investigated

The people investigated in these studies were users of happiness training techniques. Most of them participated voluntarily and were recruited via websites, flyers, and using snowball sampling methods. Recruitment focused on students, patients, and working peoples. This resulted in self-selected samples, not random samples of all users of happiness training techniques. In our definition of self-selecting, we do not strictly refer to people who truly volunteered, but also to samples of college students who participated in the research for a study credit. Several studies forced people to take part in a happiness training technique, typically in the context of an educational course or as a therapy.

#### Notation of Findings

Observed effects of happiness training were summarized in a standard format and terminology on electronic finding pages, together with methodological characteristics of the study. Veenhoven (2019f) offers a description of that process.

### Analysis

#### Organization of the Findings

We sorted the selected happiness training techniques by the nature of the intervention. The results are presented in Table 2^1^. Some of these techniques appear in more than one cell of the table. This is the case for a training that fits more than one category. The full classification of different natures and modes can be found on the WDH website^2^, (Veenhoven, 2019c).

**TABLE 2:** Classification of happiness trainings in the World Database of Happiness, collection of Correlational Findings, subject section Health: Psychological Treatment sub-subjects and number of finding-pages by December 2019.

Subject name	Number of finding pages^1^
**Happiness training**	
Best possible self-exercise	2
Cognitive reframing	1
Comparison with the happiness of similar people	1
Enlightenment about happiness	5
Goal setting training	5
Laughter yoga	1
List and practice perceived ways to happiness	1
• Practice retrospective sources of happiness	1
• Practice one’s values	2
Meditation, mindfulness	7
Novelty trying	1
Life style awareness training	1
• Life-review exercise	2
• Mood awareness training	4
Positive thinking training	3
• Count blessings/curses	7
• Gratitude training	10
• Kindness training	2
• Recall of positive events	5
Savouring training	1
Trainings for multiple mental skills	8
• Enlightenment + exercises	5
**Total**	**74**

*^1^A finding-page can report more than one separate research findings, such as obtained with different measures of happiness, different statistics and separate findings for experimental and control groups.*

#### Presentation of the Findings

In Table 3, we summarize the observed effects of happiness training on happiness using three possible signs: **+** for a positive relationship, – for a negative relationship, and 0 for a non-relationship. Statistical significance is indicated by printing the sign in **bold** (*p* < 0.05). Some of the findings are presented in a string of signs, for example, **+/+** for studies that used more than one measure of happiness, or for studies that used more than one control group. In Table 4, we present the same data quantified in effect sizes as the change in percentage of the theoretical scale range of the happiness measure used. This method allows comparison across measures with different scale ranges and is relatively easy to interpret for a general public (Borenstein et al., 2009). In Tables 5–10, we summarize the same findings, sorted by different subgroups.

**TABLE 3:** 179 research findings on the effect of happiness trainings on happiness observed direction of change and statistical significance.

Nature of happiness training	Cross-sectional had training versus did not have training	Longitudinal before versus after training			
		
		Change in treatment group only		Difference with change in control group	
			
		Post-intervention	After follow-up	Post-intervention	After follow-up
****Single kind of training****					
Best possible self-exercise		+	+	+	−
Cognitive reframing		+		+	
Comparison with similar people		+			
Enlightenment about happiness		+ +	+	−	
Goal-setting training		+/+ + − + +	+/+ + + +	+/+ + + 0 0	− 0
Laughter yoga		+		+	
List and practice perceived ways to happiness		+/+			
Practice retrospective sources of happiness		+		+	
Live up to one’s values		++	+	+ +	+
Meditation, mindfulness	+/−	+ + + + +	+ + +	+ − + +	− +
Novelty trying		+	+	0	0
Lifestyle awareness training		+	+	+	+
Life-review exercise		+		+	−/−/0
Mood awareness training		+ +/+/+	+/+ +/+/+	+/+/+	+/+/+
Positive thinking training		+ + +	+	− + +	−
• Count blessings and curses	+/0 + 0 −	+ + +	+	+	
• Gratitude training	+ − +/+ +	+ + +	−	− +	−
• Kindness training	+/+	+/−		+/−	
• Recall of positive events		+ + + +	+ +	+ +	
Savoring training		+	+	+/+	−/−
****Multiple kinds combined****					
Training of multiple mental skills	+/0	+ + + + + + + +/+	+ + + + + +/+	+ + + + + +/+	+ + + − +/+
• Enlightenment + exercises		+ +/+		+ +/+/+ +/+/+ +/+	

% independent studies positive					
- all positive	56%	96%	96%	87%	47%
- positive and significant (p < 0.05)	11%	49%	52%	37%	29%

**Signs link to finding page in*World Database of Happiness. *Click linked sign to view the page. Signs indicate observed differences. + = positive difference, significant. + = positive difference, not significant. 0 = no difference. − = negative difference, not significant. .− = negative difference, significant.**

**TABLE 4:** 150 research findings on the effect of happiness trainings on happiness in 55 studies effect sizes expressed in % change on scale range.

Nature of happiness training	Method of investigation				
	
	Cross-sectional had training versus did not have training	Longitudinal before versus after training			
		
		Change in treated group only		Difference with change in control group	
			
		Post-intervention	After follow-up	Post-intervention	After follow-up
***Single kind of training***					
Best-possible-self exercise		+5.1	+1.6	+0.5	−2.3
Cognitive reframing		+13.7		+12.5	
Enlightenment about happiness		+0.8 +3.9	+3.2	−2.9	
Goal-setting training		+13.4/+10.4c +2.9 +3.6 +5.1a −1.3	+11.9/+10.4c +1.1 +1.6a +3,6	+8.6/+9.5c +0.5a +4.3 0 0	−2.3a 0
Laughter yoga		+8.6		+8.6	
List and practice perceived ways to happiness		+0.6/+7.2c			
Practice retrospective sources of happiness		+12.5		+12.5	
Live up to one’s values		+1.0 +4.0	+2.5	+5.0 +7.8	+5.8
Meditation, mindfulness	+2.9	+4.1 +13.2 +4.6 +18.0 +8.7	+5.0 +5.6 +17.6	+8.0 −0.6 +18.0 +9.0	−0.7 +16.7
Novelty trying		+3.6a	+1.1a	0	0
Life-review exercise					−0.9/-4.6/0c
Positive thinking training		+2.4 +13.7a +4.0	+7.0	−0.4 +12.5a +5.0	−2.9
• Count blessings and curses	+6.5 0 −3.3a	+0.7 +2.4a	+1.5		
• Gratitude training	+12.3 −3.3 +14.0/+7.9c +5.4	+1.5 +2.4	−0.7	−0.8	−0.2
• Kindness training	+13.3/+6.0c	+0.6/−2.9c		+3.6/−4.0c	
• Recall of positive events		+1.9 +0.7a +3.0 +4.9	+3.5 +1.5a	+4.0 +3.3	
Savoring training		+1.3	+0.3	+1.6/+1.1c	−0.2/-0.1c
***Multiple kinds combined***					
Training of multiple mental skills		+19.5 +10.0 +6.7 +3.7 +3.6 +7.8 +5.9 +15.2/+14.8c	+13.9 +30.8 +15.8 +0.4 +8.5 +11.8/+14.8c	+29.0b +8.3 +3.4 +2.0 +3.5 +23.8/+23.4b	+27.9b +10.4 +10.4 −10.8 +23.4/+25.4b
• Enlightenment + exercises		+4.0 +7.8/+19.6c		+3.9 +8.7/+10.9/+18.5c +3.6/+3.0/+10.1c +21.6/+46.1b	

**Number of studies and participants**					
Number of studies	8	39	22	31	14
Participants (n)	630	3,539	2,126	3,562d	1,664d
Mean n	79	91	97	115	119
Median n; range	61; 3–192	43; 10–606	53; 10–606	73; 23–631	89; 37–360

**Results**					
Mean change	+5.6%	+6.0%	+7.7%	+4.7%	+1.8%
Median change	+6.0%	+4.0%	+4.3%	+3.9%	−0.1%

**Numbers denote % change on scale range and link to a finding page in*World Database of Happiness. *Use control+click to view the page. a: Not included in calculation of mean/median because study appears twice in a column. b: Not included in calculation of mean/median because of a huge decline in happiness in the control group (treated as outliers). c: Average value of multiple measures/multiple comparison groups used in calculation of mean/median. d: Numbers of participants and controls added up.**

**TABLE 5:** 66 research findings from 13 studies on the effect of happiness trainings on happiness, only studies with control group and follow-up measurement effect sizes expressed in % change on scale range.

Nature of happiness training	Method of investigation				
	
	Longitudinal before versus after training				
	
	Change in treated group only		Difference with change in control group		Follow-up time
			
	Post-intervention	After follow-up	Post-intervention	After follow-up	
Best-possible-self exercise	+5.1	+1.6	+0.5	−2.3	2 months
Goal-setting training	+3.6 +5.1a	+1.1 +1.6a	0 +0.5a	0 −2.3a	2 weeks
∙ Live up to one’s values	+4.0	+2.5	+7.8	+5.8	2 weeks
Meditation, mindfulness	+4.6	+5.6	−0.6	−0.7	6 weeks
Novelty trying	+3.6a	+1.1a	0a	0a	
Mood awareness training	+3.9/+3.7/+0.7c	+4.2/+3.7/+1.2c	+2.0/+4.0/+0.7c	+0.4/+1.1/+1.1c	2 weeks
Positive thinking training	+2.4	+7.0	−0.4	−2.9	6 months
∙ Gratitude training	+1.5	−0.7	−0.8	−0.2	2 months
Savoring training	+1.3	+0.3	+1.6/+1.1	−0.2/-0.1c	1 month
Training of multiple mental skills	+19.5 +10.0 +6.7 +3.7 +15.2/+14.8c	+13.9 +30.8 +15.8 +0.4 +11.8/+14.8c	+29.0b +8.3 +3.4 +2.0 +23.8/+23.4b	+27.9b +10.4 +10.4 −10.8 +23.4/+25.4b	6 months/1 year 1 year/3 months 4 weeks

**Number of studies and participants**					
Number of studies	13	13	11	11	
Participants (n)	945	933	1,441d	1,416d	
Mean n	73	72	131	129	
Median n; range	43; 10–306	43; 10–306	79; 37–360	79; 37–360	

**Results**					
Mean change	+6.2%	+7.3%	+2.2%	+0.9%	
Median change	+4.0%	+3.0%	+1.4%	+0.2%	

**Percentages change in upper part of the table link to finding page in*World Database of Happiness. *Click linked percentages in upper part of the table to view the page. a: not included in calculation of mean/median because study appears twice in column. b: Not included in calculation of mean/median because of huge decline in happiness in control group. c: Average effect of multiple measures in study used in calculation of mean/median. d: Numbers of participants and controls added up.**

**TABLE 6:** Observed % change in happiness by nature of happiness training: single or multiple nature training Table 4 with nature of happiness training indicated in colors.

Nature of happiness training	Method of investigation				
	
	Cross-sectional had training versus did not have training	Longitudinal before versus after training			
		
		Change in treated group only		Difference with change in control group	
			
		Post-intervention	After follow-up	Post-intervention	After follow-up
***Single kind of training***					
Best-possible-self exercise		+5.1	+1.6	+0.5	−2.3
Cognitive reframing		+13.7a		+12.5a	
Enlightenment about happiness		+0.8 +3.9	+3.2	−2.9	
Goal-setting training		+13.4/+10.4c +2.9 +3.6 +5.1a −1.3	+11.9/+10.4c +1.1 +1.6a +3.6	+8.6/+9.5c +0.5a +4.3 0 0	−2.3a 0
Laughter yoga		+8.6		+8.6	
List and practice perceived ways to happiness		+0.6/+7.2c			
Practice retrospective sources of happiness		+12.5		+12.5	
Live up to one’s values		+1.0 +4.0	+2.5	+5.0 +7.8	+5.8
Meditation, mindfulness	+2.9	+4.1 +13.2 +4.6 +18.0 +8.7	+5.0 +5.6 +17.6	+8.0 −0.6 +18.0 +9.0	−0.7 +16.7
Novelty trying		+3.6a	+1.1a	0	0
Life-review exercise					−0.9/−4.6/0c
Mood awareness training		+3.9/+3.7/+0.7c	+4.2/+3.7/+1.2c +18.9/+23.2c	+2.0/+4.0/+0.7c	+0.4/+1.1/+1.1c
Positive thinking training		+2.4 +13.7 +4.0	+7.0	−0.4 +12.5 +5.0	−2.9
• Count blessings and curses	+6.5 0 −3.3a	+0.7 +2.4a	+1.5		
• Gratitude training	+12.3 −3.3 +14.0/+7.9c +5.4	+1.5 +2.4	−0.7	−0.8	−0.2
• Kindness training	+13.3/+6.0c	+0.6/−2.9c		+3.6/−4.0c	
• Recall of positive events		+1.9 +0.7a +3.0 +4.9	+3.5 +1.5a	+4.0 +3.3	
Savoring training		+1.3	+0.3	+1.6/+1.1c	-0.2/-0.1c
***Multiple kinds combined***					
Training of multiple mental skills		+19.5 +10.0 +6.7 +3.7 +3.6 +7.8 +5.9 +15.2/+14.8c	+13.9 +30.8 +15.8 +0.4 +8.5 +11.8/+14.8c	+29.0b +8.3 +3.4 +2.0 +3.5 +23.8/+23.4b	+27.9b +10.4 +10.4 −10.8 +23.4/+25.4b
• Enlightenment + exercises		+4.0 +7.8/+19.6c		+3.9 +8.7/+10.9/+18.5c +3.6/+3.0/+10.1c +21.6/+46.1b	

Participants and results*					

All studies					
Nr of studies (n)	8 (630)	39 (3,539)	22 (2,126)	31 (3,562d)	14 (1,664d)
Mean change	+5.6%	+6.0%	+7.7%	+4.7%	+1.8%
Single nature					
Nr of studies (n)	8 (630)	29 (2,549)	16 (1,647)	24 (2,594d)	11 (1,146d)
Mean change	+5.6 %	+4.9 %	+5.4 %	+4.4 %	+1.4 %
Multiple kinds					
Nr of studies (n)	0	10 (990)	6 (479)	7 (968d)	3 (518d)
Mean change		+9.0%	+13.8%	+5.7%	+3.3%

*Table 4 with nature of happiness training indicated in colors. Numbers denote % change on scale range and link to a finding page in*World Database of Happiness. Click percentages change in upper part of the table to view the page. a: Not included in calculation of mean/median because study appears twice in column. b: Not included in calculation of mean/median because of huge decline in happiness in control group (treated as outliers). c: Average value of multiple measures/multiple comparison groups used in calculation of mean/median. d: Numbers of participants and controls added up *For detailed information on mean/median/range of sample size (see Supplementary Material).

**TABLE 7:** Observed % change of happiness by mode of the training: online, offline Table 4 with mode of happiness training indicated in colors.

Nature of happiness training	Method of investigation				
	
	Cross-sectional had training versus did not have training	Longitudinal before versus after training			
		
		Change in treated group only		Difference with change in control group	
			
		Post-intervention	After follow-up	Post-intervention	After follow-up
***Single kind of training***					
Best-possible-self exercise		+5.1	+1.6	+0.5	−2.3
Cognitive reframing		+13.7a		+12.5a	
Enlightenment about happiness		+0.8 +3.9	+3.2	−2.9	
Goal-setting training		+13.4/+10.4c +2.9 +3.6 +5.1a -1.3	+11.9/+10.4c +1.1 +1.6a +3.6	+8.6/+9.5c +0.5a +4.3 0 0	−2.3a 0
Laughter yoga		+8.6		+8.6	
List and practice perceived ways to happiness		+0.6/+7.2c			
Practice retrospective sources of happiness		+12.5		+12.5	
Live up to one’s values		+1.0 +4.0	+2.5	+5.0 +7.8	+5.8
Meditation, mindfulness	+2.9	+4.1 +13.2 +4.6 +18.0 +8.7	+5.0 +5.6 +17.6	+8.0 −0.6 +18.0 +9.0	−0.7 +16.7
Novelty trying		+3.6a	+1.1a	0	0
Life-review exercise					−0.9/-4.6/0
Mood awareness training		+3.9/+3.7/+0.7c	+4.2/+3.7/+1.2c +18.9/+23.2c	+2.0/+4.0/+0.7c	+0.4/+1.1/+1.1c
Positive thinking training		+2.4 +13.7 +4.0	+7.0	−0.4 +12.5 +5.0	−2.9
• Count blessings and curses	+6.5 0 −3.3a	+0.7 +2.0a	+1.5		
• Gratitude training	+12.3 −3.3 +14.0/+7.9c +5.4	+1.5 +2.4	−0.7	−0.8	−0.2
• Kindness training	+13.3/+6.0c	+0.6/−2.9c		+3.6/−4.0c	
• Recall of positive events		+1.9 +0.7a +3.0 +4.9	+3.5 +1.5a	+4.0 +3.3	
Savoring training		+1.3	+0.3	+1.6/+1.1c	−0.2/-0.1c
***Multiple kinds combined***					
Training of multiple mental skills		+19.5 +10.0 +6.7 +3.7 +3.6 +7.8 +5.9 +15.2/+14.8c	+13.9 +30.8 +15.8 +0.4 +8.5 +11.8/+14.8c	+29.0b +8.3 +3.4 +2.0 +3.5 +23.8/+23.4b	+27.9b +10.4 +10.4 −10.8 +23.4/+25.4b
• Enlightenment + exercises		+4.0 +7.8/+19.6c		+3.9 +8.7/+10.9/+18.5c +3.6/+3.0/+10.1c +21.6/+46.1b	

Participants and results*					

All studies					
Nr of studies (n)	8 (630)	39 (3,539)	22 (2,126)	31 (3,562d)	14 (1,664d)
Mean change	+5.6%	+6.0%	+7.7%	+4.7%	+1.8%
Online e-training					
Nr of studies (n)	0	4 (735)	2 (275)	1 (349d)	1 (349d)
Mean change		+5.3%	+12%	+2.2%	+0.9%
Offline guided training					
Nr of studies (n)	8 (630)	35 (2,804)	20 (1,851)	30 (3213d)	13 (1,315d)
Mean change	+5.6%	+6.0%	+7.3%	+4.8%	+1.9%

*Table 4 with mode of happiness training indicated in colors. Numbers denote % change on scale range and link to a finding page in*World Database of Happiness. Use control+click to view the page. a: Not included in calculation of mean/median because study appears twice in a column. b: Not included in calculation of mean/median because of huge decline in happiness in control group (treated as outliers). c: Average value of multiple measures/multiple comparison groups used in calculation of mean/median. d: Numbers of participants and controls added up. *For detailed information on mean/median/range of sample size) (see Supplementary Material).

**TABLE 8:** Observed change of happiness by context of the training Table 4 with context of happiness training indicated in colors.

Nature of happiness training	Method of investigation				
	
	Cross-sectional had training versus did not have training	Longitudinal before versus after training			
		
		Change in treated group only		Difference with change in control group	
			
		Post-intervention	After follow-up	Post-intervention	After follow-up
***Single kind of training***					
Best-possible-self exercise		+5.1	+1.6	+0.5	−2.3
Cognitive reframing		+13.7a		+12.5a	
Enlightenment about happiness		+0.8 +3.9	+3.2	−2.9	
Goal-setting training		+13.4/+10.4c +2.9 **+3.6** +5.1a -1.3	+11.9/+10.4c +1.1 +1.6a +3.6	+8.6/+9.5c +0.5a +4.3 0 0	−2.3a 0
Laughter yoga		**+8.6**		**+8.6**	
List and practice perceived ways to happiness		+0.6/**+7.2c**			
Practice retrospective sources of happiness		+12.5		+12.5	
Live up to one’s values		+1.0 +4.0	+2.5	+5.0 +7.8	+5.8
Meditation, mindfulness	+2.9	**+4.1** +13.2 **+4.6** +18.0 **+8.7**	**+5.0** **+5.6** +17.6	+8.0 −0.6 +18.0 **+9.0**	−0.7 +16.7
Novelty trying		+3.6a	+1.1a	0	0
Life-review exercise					−0.9/-4.6/0
Mood awareness training		**+3.9/+3.7/+0.7c**	**+4.2/+3.7/+1.2c +18.9/+23.2c**	+2.0/+4.0/+0.7c	+0.4/+1.1/+1.1c
Positive thinking training		+2.4 +13.7 **+4.0**	+7.0	−0.4 +12.5 **+5.0**	−2.9
• Count blessings and curses	+6.5 0 −3.3a	+0.7 +2.4a	+1.5		
• Gratitude training	+12.3 −3.3 +14.0/+7.9c +5.4	+1.5 +2.4	−0.7	−0.8	−0.2
• Kindness training	+13.3/+6.0c	+0.6/−2.9c		+3.6/−4.0c	
• Recall of positive events		+1.9 +0.7a +3.0 +4.9	+3.5 +1.5a	+4.0 +3.3	
Savoring training		+1.3	+0.3	+1.6/+1.1c	−0.2/-0.1c
***Multiple kinds combined***					
Training of multiple mental skills		+19.5 **+10.0** **+6.7** **+3.7** +3.6 **+7.8** **+5.9** +15.2/+14.8c	+13.9 **+30.8** **+15.8** **+0.4** +8.5 +11.8/+14.8c	+29.0b **+8.3** +3.4 +2.0 +3.5 +23.8/+23.4b	+27.9b **+10.4** +10.4 −10.8 +23.4/+25.4b
• Enlightenment + exercises		+4.0 +7.8/+19.6c		+3.9 +8.7/+10.9/+18.5c +3.6/+3.0/+10.1c +21.6/+46.1b	

Participants and results*					

All studies					
Nr of studies (n)	8 (630)	39 (3,539)	22 (2,126)	31 (3.562d)	14 (1,664d)
Mean change	+5.6%	+6.0%	+7.7%	+4.7%	+1.8%
Care setting					
Nr of studies (n)	3 (127)	2 (56)	2 (107)	2 (103d)	1 (152d)
Mean change	+11%	+12.6%	+14.4%	+8.5%	+16.7%
Education setting					
Nr of studies (n)	2 (322)	15 (1,623)	7 (953)	14 (2,011d)	4 (283d)
Mean change	+1.6%	+4.8%	+3.6%	+5.0%	+0.2%
Mean % of change	+1.6	+4.8	+3.6	+5.0	+0.2
Work setting					
Nr of studies (n)	0	2 (64)	3 (163)	0	0
Mean change		+17. %	+10.3%		

*Table 4 with context of happiness training indicated in colors. Numbers denote % change on scale range and link to a finding page in*World Database of Happiness. Click percentages change in upper part of the table to view the page. a: Not included in calculation of mean/median because study appears twice in a column. b: Not included in calculation of mean/median because of huge decline in happiness in control group (treated as outliers). c: Average value of multiple measures/multiple comparison groups used in calculation of mean/median. d: numbers of participants and controls added up. *For detailed information on mean/median/range of sample size (see Supplementary Material).

**TABLE 9:** Observed change of happiness by mode of the training: participation voluntary, paid, obligatory Table 4 with mode of happiness training indicated in colors.

Nature of happiness training	Method of investigation				
	
	Cross-sectional had training versus did not have training	Longitudinal before versus after training			
		
		Change in treated group only		Difference with change in control group	
			
		Post-intervention	After follow-up	Post-intervention	After follow-up
***Single kind of training***					
Best-possible-self exercise		+5.1	+1.6	+0.5	-2.3
Cognitive reframing		+13.7a		+12.5a	
Enlightenment about happiness		+0.8 +3.9	+3.2	−2.9	
Goal-setting training		+13.4/+10.4c +2.9 +3.6 +5.1a -1.3	+11.9/+10.4c +1.1 +1.6a +3.6	+8.6/+9.5c +0.5a +4.3 0 0	-2.3a 0
Laughter yoga		+8.6		+8.6	
List and practice perceived ways to happiness		+0.6/+7.2c			
Practice retrospective sources of happiness		+12.5		+12.5	
Live up to one’s values		+1.0 +4.0	+2.5	+5.0 +7.8	+5.8
Meditation, mindfulness	+2.9	+4.1 +13.2 +4.6 +18.0 +8.7	+5.0 +5.6 +17.6	+8.0 −0.6 +18.0 +9.0	−0.7 +16.7
Novelty trying		+3.6a	+1.1 a	0	0
Life-review exercise					−0.9/-4.6/0
Mood awareness training		+3.9/+3.7/+0.7c	+4.2/+3.7/+1.2c +18.9/+23.2c	+2.0/+4.0/+0.7c	+0.4/+1.1/+1.1c
Positive thinking training		+2.4 +13.7 +4.0	+7.0	−0.4 +12.5 +5.0	−2.9
• Count blessings and curses	+6.5 0 −3.3	+0.7 +2.4a	+1.5		
• Gratitude training	+12.3 −3.3a +14.0/+7.9c +5.4	+1.5 +2.4	−0.7	−0.8	−0.2
• Kindness training	+13.3/+6.0c	+0.6/−2.9c		+3.6/−4.0c	
• Recall of positive events		+1.9 +0.7a +3.0 +4.9	+3.5 +1.5a	+4.0 +3.3	
Savoring training		+1.3	+0.3	+1.6/+1.1c	−0.2/-0.1c
***Multiple kinds combined***					
Training of multiple mental skills		+19.5 +10.0 +6.7 +3.7 +3.6 +7.8 +5.9 +15.2/+14.8c	+13.9 +30.8 +15.8 +0.4 +8.5 +11.8/+14.8c	+29.0b +8.3 +3.4 +2.0 +3.5 +23.8/+23.4b	+27.9b +10.4 +10.4 −10.8 +23.4/+25.4b
• Enlightenment + exercises		+4.0 +7.8/+19.6c		+3.9 +8.7/+10.9/+18.5c +3.6/+3.0/+10.1c +21.6/+46.1b	

Participants and results*					

All studies					
Nr of studies (n)	8 (630)	39 (3,539)	22 (2,126)	31 (3,562d)	14 (1,664d)
Mean change	+5.6%	+6.0%	+7.7%	+4.7%	+1.8%
Voluntary					
Nr of studies (n)	7 (500)	24 (1,991)	15 (1,245)	17 (2,023d	10 (1,440d)
Mean change	+6.8%	+6.4%	+7.8%	+4.0%	+1.2%
Paid/study credit					
Nr of studies (n)	0	5 (122)	2 (49)	5 (253d)	2 (95d)
Mean change		+4.4%	+16.7%	+6.1%	+8.1%
Mandatory					
Nr of studies (n)	1 (130)	10 (1,426)	5 (832)	9 (1,286d)	2 (129d)
Mean change	−3.3%	+5.7%	+3.9%	+5.2%	−1.6%

*Table 4 with mode of happiness training indicated in colors. Numbers denote % change on scale range and link to a finding page in*World Database of Happiness. Click percentages change in upper part of the table to view the page. a: Not included in calculation of mean because study appears twice in table. b: Not included in calculation of mean because of huge decline in happiness in control group (treated as outliers). c: Average value of multiple measures/multiple comparison groups used in calculation of mean. d: Numbers of participants and controls added up. *For detailed information on mean/median/range of sample size (see Supplementary Material).

**TABLE 10:** Size of changes in happiness among different users: children, university students, elderly Table 4 with users indicated in colors.

Nature of happiness training	Method of investigation				
	
	Cross-sectional had training versus did not have training	Longitudinal before versus after training			
		
		Change in treated group only		Difference with change in control group	
			
		Post-intervention	After follow-up	Post-intervention	After follow-up
***Single kind of training***					
Best-possible-self exercise		+5.1	+1.6	+0.5	−2.3
Cognitive reframing		+13.7a		+12.5a	
Enlightenment about happiness		+0.8 +3.9	+3.2	−2.9	
Goal-setting training		+13.4/+10.4c +2.9 +3.6 +5.1a -1.3	+11.9/+10.4c +1.1 +1.6a +3.6	+8.6/+9.5c +0.5a +4.3 0 0	−2.3a 0
Laughter yoga		+8.6		+8.6	
List and practice perceived ways to happiness		+0.6/+7.2c			
Practice retrospective sources of happiness		+12.5		+12.5	
Live up to one’s values		+1.0 +4.0	+2.5	+5.0 +7.8	+5.8
Meditation, mindfulness	+2.9	+4.1 +13.2 +4.6 +18.0 +8.7	+5.0 +5.6 +17.6	+8.0 −0.6 +18.0 +9.0	−0.7 +16.7
Novelty trying		+3.6a	+1.1a	0	0
Life-review exercise					−0.9/-4.6/0
Mood awareness training		+3.9/+3.7/+0.7c	+4.2/+3.7/+1.2c +18.9/+23.2c	+2.0/+4.0/+0.7c	+0.4/+1.1/+1.1c
Positive thinking training		+2.4 +13.7 +4.0	+7.0	−0.4 +12.5 +5.0	−2.9
• Count blessings and curses	+6.5 0 −3.3a	+0.7 +2.4a	+1.5		
• Gratitude training	+12.3 −3.3 +14.0/+7.9c +5.4	+1.5 +2.4	−0.7	−0.8	−0.2
• Kindness training	+13.3/+6.0c	+0.6/−2.9c		+3.6/−4.0c	
• Recall of positive events		+1.9 +0.7a +3.0 +4.9	+3.5 +1.5a	+4.0 +3.3	
Savoring training		+1.3	+0.3	+1.6/+1.1c	−0.2/-0.1c
***Multiple kinds combined***					
Training of multiple mental skills		+19.5 +10.0 +6.7 +3.7 +3.6 +7.8 +5.9 +15.2/+14.8c	+13.9 +30.8 +15.8 +0.4 +8.5 +11.8/+14.8c	+29.0b +8.3 +3.4 +2.0 +3.5 +23.8/+23.4b	+27.9b +10.4 +10.4 −10.8 +23.4/+25.4b
• Enlightenment + exercises		+4.0 +7.8/+19.6c		+3.9 +8.7/+10.9/+18.5c +3.6/+3.0/+10.1c +21.6/+46.1b	

Participants and results*					

All studies					
Nr of studies (n)	8 (630)	39 (3,539)	22 (2,126)	31 (3,562d)	14 (1,664d)
Mean change	+5.6%	+6.0%	+7.7%	+4.7%	+1.8%
Children					
Nr of studies (n)	1(130)	4 (1,051)	3 (670)	3 (760d)	2 (129d)
Mean change	−3.3%	+1.4%	+2.6%	−1.4%	−1.5%
University students					
Nr of studies (n)	2 (297)	19 (1,370)	8 (840)	20 (2,251d)	8 (1,100d)
Mean change	+3.7%	+5.0%	+8.2%	+4.8%	+1.9%
Elderly					
Nr of studies (n)	0	1 (88)	1 (88)	0	0
Mean change		+1.9%	+3.5%		

*Table 4 with users indicated in colors. Numbers denote % change on scale range and link to a finding page in*World Database of Happiness. Click percentages change in upper part of the table to view the page. a: Not included in calculation of mean because study appears twice in table. b: Not included in calculation of mean because of huge decline in happiness in control group (treated as outliers). c: Average value of multiple measures/multiple comparison groups used in calculation of mean. d: Numbers of participants and controls added up. *For detailed information on mean/median/range of sample size (see Supplementary Material).

#### Links to Online Detail

**FIGURE 3:**
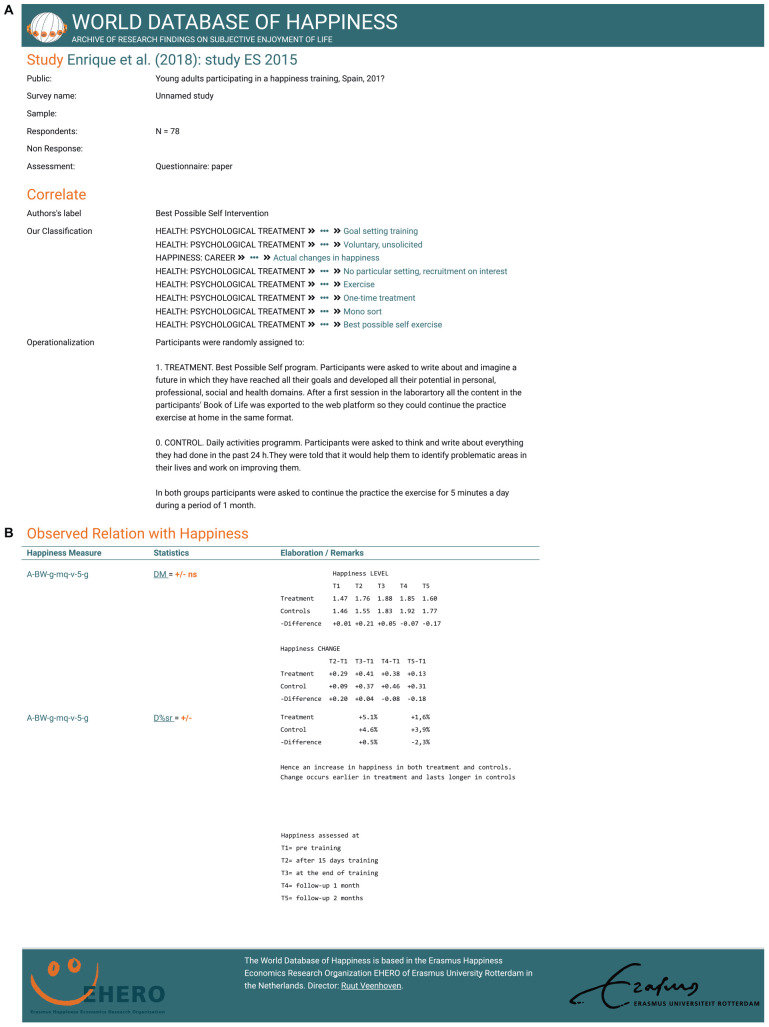
Example of a findings page in the World Database of Happiness Correlational finding on Happiness and Best-Possible-Self exercise; Subject code: H16ad03a.

As noted above, the World Database of Happiness is a collection of “finding pages” on which the results of empirical research on happiness are reported in a standard language and format. An example of a findings page is presented in Figure 3. In this paper we use links to such online finding pages. All the signs in Tables 3–10 link to finding pages in the World Database of Happiness, which serves as an online appendix for this article. If you click on a sign, the corresponding finding page will open and offer details of the observed relationship, such as on the people investigated, the sampling method used, the training technique, the happiness measure, and the statistical analysis. This technique allows us to present the main trends in the findings, while keeping this paper to a controllable size and, at the same time, allowing the readers to check detail.

#### Indicators of Effect

We considered three indicators of the effect of following a happiness training. A first indicator was the difference in happiness among people who have taken a happiness training course and people who have not. An evident weakness of this cross-sectional approach is that selectiveness can play us false, in particular because users of happiness training techniques are likely to be less happy than non-users.

A better indicator of how well a happiness training technique has worked is the change in happiness observed among users of a happiness training technique, both pre-test to post-test and in the long-term follow-up. A weakness of this longitudinal approach is that the observed effect may be due to being part of an intervention as such rather than its content. In clinical psychology this is known as the “placebo effect” and in industrial psychology as the “Hawthorne effect” (Franke and Kaul, 1978).

This problem can be solved using a control group that gets an equally credible intervention with another content or a place on a waiting list. The change in happiness of an intervention group compared with a change in a control group was our third indicator. It should be noted that the control groups used in the studies were heterogeneous, which makes the interpretation of the difference more complicated (Dickens, 2017).

### Advantages and Disadvantages of Using an Online Finding Archive

There are pros and cons to the use of a findings archive such as the World Database of Happiness and plusses and minuses to the use of links to an online source.

#### Advantages

(1) Efficient gathering of research on happiness. (2) Sharp conceptual focus fitting a specific definition of happiness. (3) Uniform description of research findings. (4) Storage of findings pages in an easily searchable and freely available database. (5) Online availability of the database and the separate finding pages it contains. 6) Presentation of the available research findings in easy-to-view tables using links to online finding pages. (7) The technique is useful for ongoing harvesting of research findings on a particular subject. It is easy to update review papers of this kind by entering new signs in the tables.

#### Disadvantages

(1) The sharp conceptual focus cannot easily be changed. (2) Considerable investment is required to keep the archive up to date. (3) Links to online finding pages work only for electronic texts. (4) The standard information available in the excerpts is not always sufficient for judging the methodological quality, which is a hindrance, specifically, for the purposes of our research as links to the full text of the original research reports are required. (5) Using a finding archive such as the World Database of Happiness speeds up the process of finding all the relevant studies, but this comes at the cost of less control when determining the scope of the review and the questions it will address. (6) The new presentation may strike some readers as unfamiliar; the technique is not yet known under a particular name and not yet described in textbooks.

### Differences With Meta-Analysis

Our research synthesis lacks three practices common in statistically more sophisticated meta-analysis. (1) Given the number and heterogeneity of the available findings, we did not assess statistical significance from the field taken as a whole. We restricted our findings to mentioning the percentage of significant results in each of the columns of Table 3 separately. For the same reason, we did not quantify the effects of moderator variables but presented these visually in Tables 6–10. (2) We did not correct for sample size because the large number of the online interventions would have out crowded the fewer face-to-face trainings. (3) We did not take variance into account when calculating the average effect size using independent findings. We used an average “raw” percentage of the change in scale range instead. This is a consequence of the conceptual rigor in measuring the affective component of happiness. Almost half of the studies in the analysis used an affect balance scale, such as the PANAS (Watson et al., 1988), and reported the change in average positive and negative affect and the accompanying standard deviations separately, if at all. Yet, in isolation these scores do not fit our concept of hedonic level. You can experience much negative affect, but still be happy because you experience even more positive affect. Only the “Affect Balance Scores” are used in our analysis and, for that reason, we computed affect balance scores ourselves, subtracting reported average negative affect from average positive affect. This gave us group averages, but no individual scores and no information on the spread. This left us with effect sizes that can be described as “raw,” in the sense that the dispersion around the mean is not taken into account. We sacrificed some of the statistical sophistication of common meta-analysis to report on as much data as possible that fits our definition of happiness. The advantage is that the effect size as a simple average change on the scale range used is intuitively meaningful (Borenstein et al., 2009).

Alongside these differences due to data availability, this research synthesis differs from common meta-analysis in the following ways: (1) The use of links to online finding pages provides the reader with far more detail than that which can be offered by standard reports of meta-analysis, while the standardized descriptions on the finding pages will also provide the analyst with a closer look at the research findings to be synthesized. (2) The presentation of research finding in tabular schemes, such as in Table 3, provides the reader with a visual overview from which the pattern of results can easily be recognized and from which differences in research methods used strike the eye. Moderators can also be visualized, using variant as of such tables in which differences in population or happiness measures are indicated using colors, as the reader will see in Tables 6–10. (3) As such, the method applied here tells the reader more about these heterogeneous and incomplete data than a common meta-analysis could have done with a reduction in numbers. (4) This research synthesis is conceptually more focused than the research syntheses of the effect of PPIs on “well-being” mentioned in section “Research Questions,” and, as such, it generates new information.

### Publication Bias

We study the distribution of effect sizes and perform a p-curve analysis to detect possible selective publishing and p-hacking of empirical studies that may have influenced our results.

## Results

Let us now revert to the research questions mentioned in section “Concepts of Happiness” and answer them one by one. Note that many of these results are tentative because of the limited availability of data.

### Do Happiness Training Techniques Add to Happiness?

The answer to this question is presented in Table 3 on which **+** and – signs indicate whether or not the use of training had added to the average happiness of its users. In the table, we see mainly plusses, accounting for 149 of 179 or, roughly, 83%. About half of these denote statistically significant effects as indicated in **bold**. The percentage of 83% is based on all findings and not just on independent studies. Taking a closer look and only counting independent studies in each of the columns in Table 3, we see in the bottom row that the proportion of studies with solely positive outcomes is highest (96%) among studies that compared happiness before and after training and at follow-up, and lowest (56%) among the cross-sectional studies, while the proportion of positive studies that compared the change in happiness to a control group is 87% post-intervention and 47% after follow-up. About half of the positive studies reported a statistically significant (*p* < 0.05) rise in happiness. So far, the available findings suggest that happiness training techniques typically work. A further first impression from Table 3 is that effects do not differ very much across different kinds of happiness training.

### How Strong Is the Effect of Following Happiness Training on Later Happiness?

An answer to this question is found in Table 4, in which the sizes of the observed changes in happiness following happiness training are presented. Table 4 follows the same format as Table 3 but adds observed effect sizes to the + and – signs as much as the available data allow. We computed effect sizes as a percentage of the scale range (D%sr) and calculated means and medians for each column of Table 4. In the table some studies appear twice because they fit two categories and some studies yielded more than one result (e.g., if more than one measure of happiness or more than one control group was used). Therefore, we calculated an average effect size for each individual study by averaging different happiness measures and/or control groups, and we did not count double studies twice. In this way, we ensured that only independent findings were used in calculating the overall mean and median effect sizes, as is recommended by Cheung (2019). We see a positive mean effect on happiness for all five indicators used, which is highest for absolute change in happiness at follow-up (+7.7%) and lowest for difference with controls at follow-up (+1.8%).

#### Happiness of People Who Followed a Training Versus Non-users

Looking at the columns in Table 4 individually, we first see the results of eight studies that compared people who had participated in happiness training with people who had not. The observed differences range between −3.3% and +14%, with an average of +5.6%. As noted above, this method is likely to underestimate the effect of participating in happiness training since unhappy people may be more likely to take training. In this context, it is worth noting that seven of these eight studies were among self-selected participants.

#### Happiness Before and After the Training

The following two columns in Table 4 contain observed changes in happiness following participation in happiness training. The first of these columns consists of the change in happiness at the start of the training (pre-test) and right after (post-test). The second column consists of the change in happiness between pre-test and later follow-up, typically some weeks or months later. The average gain in happiness is smaller right after the training (6.0%) than at follow-up (7.7%).

#### Happiness of Treated and Controls

The two right-hand columns in Table 4 consist of the findings of longitudinal studies that involved a control group and express the effect of following happiness training as the difference in change of happiness between users of such training techniques and non-users subjected to another form of intervention. Of the 36 studies included here, in just one (2.5%) participants were randomly assigned to either a treatment or control condition, and in all other studies, non-probability samples were used (40% self-selected, 35% chunk, 8% purposive, and 1% accidental; a description of these sample types is found in Veenhoven (2019f). Control conditions were most often passive, just filling out happiness questionnaires (50%). Less common were writing exercises (15%), giving information about happiness (8%), and some studies just compared different approaches to happiness, such as changing circumstances and activities.

A look at the bottom row of Table 4 shows that, on average, the treated gained more happiness than the controls, the difference at post-test being 4.7% and at follow-up 1.8%. These gains are considerably smaller than the above-mentioned change in the treated. One of the reasons is the negative effects noted with a minus sign, where the gain in happiness was found to be higher in the control group than among the treated. A closer look at these 12 findings reveals that none of the negative effects were significant and the largest change (-10.8%) was due to an unexplained increase in the happiness of the control group. The experimental group was slightly happier.

#### Happiness Changes for Studies That Used Control Groups and Presented Data Before and After Intervention and at Follow-Up

There is some heterogeneity in the studies included in these columns, since some report only a pre-post difference in happiness and other studies only the difference between pre-test and follow-up. We present a selection from the findings of Tables 4, 5, with 66 research findings from 13 studies that (1) used a control group and (2) had measurements at pre-intervention, post-intervention, and follow-up. This selection gives a fairer insight into the observed differences at different measurements. The observed differences in the change in happiness between intervention and control groups ranged between −0.8% and +29%. After the removal of two outliers with a strong negative trend in the happiness of the control group, the mean difference was 2.2% and the median 1.4%. For these studies the effects of the intervention were higher without comparison with the control. The mean intervention effect was 6.2% post-intervention and 7.3% at follow-up.

### How Long-Lasting Is the Effect?

We added a column on the length of the follow-up in Table 5, which varied between 2 weeks and one year. For follow-up periods of 2 weeks to 3 months, the observed change in happiness between pre-test and post-test is greater than the change between pre-test and follow-up, however, in two cases with a 6-month follow-up, hardly any difference was found. For the two cases of follow-up after a year, a larger change was found at follow-up than right after the training; this suggests a declining effect in the short run, but a sleeper effect in the long run.

### What Nature of Happiness Training Techniques Work Best?

The data were not sufficient to answer this question because of a lack of data for most of the categories. The study on laughter yoga had promising results, but was the only one of its kind. Here are some tentative indications.

#### Single Nature Training Techniques

The greatest changes in happiness (>10%) occurred after training that focused on either cognitive reframing, goal setting, laughter yoga, practicing retrospective sources of happiness, mood awareness training, or meditation. Most of the observed changes were smaller than 10%. Greater gains in happiness compared to a control group were only found for meditation and mood awareness training.

#### Multiple Nature Trainings

The happiness trainings that used multiple techniques stand out as having high effects on happiness, with effect sizes that are almost double the average effect of single nature training techniques. See Table 6 in which interventions using a single technique are signaled in red and the multiple techniques in blue. Part of this effect may be due to the length of the training since the application of multiple techniques typically requires more sessions. The data do not allow a view of what mix of approaches works best since most of the courses used the same set of “14 fundamentals” proposed by Fordyce (1977).

### What Modes of Trainings Work Best?

Happiness training is given in different contexts and in different ways. Differences in effects on happiness appear to be small.

#### Online Versus Offline

From Table 7 we can see that no clear picture emerged. The online trainings can be recognized in the table by the color blue and the offline trainings by the color red. Online training yields smaller effects on happiness post-test, but larger effects at follow-up, if not compared to a control group. When differences with a control group are taken into account, the online effect is smaller than that for guided interventions, both post-test and at follow-up.

#### Context of Happiness Training

Happiness trainings are provided in the institutional context of care, in educational settings, and at work, but also sought independently by people interested in raising their happiness. The latter group is discussed in the following paragraph. The observed effects on happiness for these different contexts are presented in Table 8, with the color orange indicating a care setting, the color blue indicating an educational setting, and the color green indicating a work setting. The training techniques were least effective in the educational setting. Happiness in care settings yielded higher gains in happiness. Two of the three trainings in a work context had considerable effects, but none of these studies employed a control group.

### What Kinds of People Profit Most From Using a Happiness Training?

Users of the happiness training techniques reviewed in this paper have much in common, namely that most live in an individualized modern society and most have received higher education.

#### Initially Low on Trained Characteristics

Training is likely to be more effective among people in need of improvement in their trained skills. This expectation was confirmed in three studies. Chan (2010) compared the effect of gratitude training on people who were high or low on gratitude. Not unexpectedly, the people low on gratitude experienced a significantly more positive affect after the intervention. The e-intervention by Bakker et al. (2020) yielded a greater gain in happiness among the initially least happy users. The level of happiness of the self-selected participants in this study was slightly lower than in the general population. Likewise, Coote and MacLeod (2012) report the largest change in happiness at follow-up in a depressed group at baseline.

#### Self-Selected

Happiness training techniques are likely to be more effective among people who participate voluntarily, typically because they are motivated to improve their happiness and hold positive opinions about psychological treatment. Self-selection is known to be a factor in effectiveness of psychotherapy (Le et al., 2014). We checked this expectation and show the results in Table 9, which is a variant of Table 4, but with changes in happiness among self-selected users indicated in green, changes among mandatory participants indicated in purple, and changes among paid participants indicated in blue. Indeed, happiness gains tend to be higher among voluntary participants than mandatory participants. Paid participation in the form of money or study credits also yielded favorable results, perhaps because this also can be considered a form of voluntary participation.

#### Age Differences: Children and University Students

Happiness training is likely to be more effective among users who are able to reflect on themselves and their lives and for that reason we expected greater effects among university students than among children in primary and secondary education. This expectation is confirmed by the average scores in the bottom rows of Table 10. There was only one study among elderly participants, with modest positive results. The studies with children are visible in orange, with the university students in blue and with the elderly in green.

### Publication Bias?

This review is based on published reports of effect studies and wemust be aware of the possibility of publication bias. Unwelcome research results are often not submitted for publication by investigators or are rejected by journals. The first thing to note in this context is that a substantial share of the findings in Table 3 are negative (13%) or zero (4%) and that only 40% of the findings are significant and positive. A more formal check for this “file-drawer” problem and “p-hacking” as the “sole explanation of the evidential value of a set of significant findings” is a p-curve analysis (Simonsohn et al., 2014). We performed such an analysis including 15 studies, which reported a statistically significant increase in happiness after completing a happiness training. In a p-curve analysis, only one finding from a particular study can be included. We used the follow-up measure if available and the post-treatment measure if a follow-up measure was lacking. If more than one happiness measure was reported, we chose overall happiness rather than affect balance or mixed measures. We excluded cross-sectional findings and treatment versus control comparisons because a reported difference does not necessarily mean that happiness increased or decreased within the treatment group. The statistically significant results of some studies had to be excluded from the analysis because the statistics were poorly reported. The p-curve analysis supports the presence of evidential value (*p* < 0.01 for binomial test and *Z* = -9.91, *p* < 0.001 for continuous test). Thus, selective reporting and/or p-hacking is unlikely to be the sole explanation for the significant findings included in our research synthesis.

## Discussion

What does this all mean for individuals and organizations looking for possibilities to boost happiness?

### Main Finding: Happiness Trainings Boost Happiness

Together, our findings suggest that happiness training techniques usually have positive effects on happiness and that negative effects are uncommon. This appeared to be the case for different interventions, delivered in different ways, for different groups, in different settings. This can be seen as a confirmation of a basic tenet in positive psychology – that it is worthwhile not only to solve one’s problems, but also to enhance strengths and life skills (Seligman and Csikszentmihalyi, 2000; Boniwell, 2012).

### How Strong Is the Effect?

We estimate the real average gain at follow-up to be somewhere around 5%. From the bottom rows of Tables 4, 5 we can see that gains in happiness following happiness training are smaller when the change in happiness in a control group is subtracted, the difference for all studies being 4.7% for gains at post-intervention and 1.8% at follow-up. This reduction is commonly seen as a placebo effect. But is it in this case?

Inevitably, awareness of their happiness has been raised among the controls, as they answered questions on their happiness during a pre-test, post-test, and follow-up. In the waiting list condition, the prospect of participation in happiness training will also have sharpened their awareness of their personal happiness. Below, we will see that raising awareness of how happy one feels tends to raise the level of one’s happiness, probably by its effects on the choices people make. Another thing to keep in mind is that the control groups differed considerably, which makes it difficult to interpret the differences with the experimental group (e.g., Dickens, 2017). Some only used passive controls that filled in a survey while others received an alternative treatment, which is likely to have produced a gain in happiness by itself (e.g., in the case of doing a writing exercise). Expressive writing can be healing (Pennebaker, 2018). Together, this all means that we should not just take the lowest value at the bottom rows of Tables 4, 5 to be the best estimate.

### Should a 5% Gain in Happiness Be Considered Modest or Much?

At first sight, a 5% gain in happiness does not impress as a great effect of following a happiness training. Yet, we are dealing with a small gain of a big thing – satisfaction with one’s life as a whole – which in its turn brings several other desirable things in its trail, such as a longer lifetime (Veenhoven, 2015). Next to this absolute impact on happiness of following happiness training, we can also look at its impact relative to other determinants.

#### Financial Equivalence

Analyses on the German Socio-Economic Panel Study (GSOEP) have yielded estimates of the effect of change in household income on happiness. Using this dataset over the years 2003 through 2008, Pfeifer (2013) reports that “Household income significantly increases life satisfaction on average by about 0.085 points per 1,000 Euros additional monthly net income in the pooled regressions and by about 0.039 points in the fixed effects regressions.” Departing from the latter most conservative estimate of a 0.04 point rise on the 0–10 happiness scale per 1000 Euro additional monthly income, the 5% (0.05) gain in happiness following a happiness training course equals a gain in monthly income of about €1.250, which was about one-third of the average household income in Germany at that time. Such income equivalences will differ across places, persons, and happiness variants, but are still considerable and comparable to amounts observed in studies on monetary compensation for losses in happiness, such as due to airport noise (Van Praag and Baarsma, 2005).

#### Similarity to Impact of Life Events

Bakker et al. (2020) compared the change in happiness following the use of their online Happiness Indicator with effects of real life events on happiness as observed in longitudinal studies. Getting married appears to raise happiness by some 5% and a gain of only 0.5% in happiness was found for winning a lottery. Becoming unemployed reduces happiness by 8% and the loss of one’s spouse by 12%. From this perspective, the average 5% gain in happiness after having followed a happiness training course is impressive.

### What Works for Whom?

We can only provide provisional answers to the questions about what kind(s) of training techniques work best and what types of people profit most from joining a happiness training course. Multiple interventions seem to outperform single interventions, which is in line with the meta-analysis by Hendriks et al. (2019), which reports a higher effect size than the meta-analysis for single interventions that we mentioned above. The fact that multiple interventions seem to work better may have to do with the variety that is needed to prevent hedonic adaptation after improvements (Sheldon and Lyubomirsky, 2012). Another reason may be that happiness can be thought of as a signal that people are doing well in life and that a lot of different skills are necessary to achieve this (Veenhoven, 2008).

Other results are more tentative. As expected, happiness training seemed more effective for adults, probably because of their ability to reflect on their lives. Training also worked better for people participating voluntarily. Pursuing happiness requires effort and that may be in short supply if you are forced to participate, for example, in a classroom setting (Sheldon and Lyubomirsky, 2019). Personal involvement may also explain why offline training worked better directly after the training, whereas online training yielded superior results at follow-up. Online training requires more internal motivation to complete, and this may increase the likelihood that people keep expanding their life skills after training has ended.

We should stress that these conclusions are tentative because the different aspects of what works for whom are clearly related in our set of studies. An example is that mandatory participation was often in the classroom setting and the worse results may just as well have to do with the (school) age of the participants or with the lack of free choice. A weakness of our research synthesis is that it is less suited to answer the more specific questions. We lacked statistical possibilities to untangle the influence of covariates.

There is, however, an advantage of bringing together all the research of happiness training techniques We discovered that efforts to merely raise happiness awareness were quite effective in raising happiness. This discovery is not yet sufficiently recognized by the field (Ludwigs et al., 2018). Therefore, we discuss this in a separate paragraph.

### Raising Awareness Works

Most happiness training techniques aim to foster specific life skills, such as planning for the future or seeing positive things in life. The idea behind these interventions is that such aptitudes help to make one’s life more satisfying. Evident limitations of this approach are that (1) training is functional only when skills can be meaningfully improved, and (2) that the functionality of particular skills may not fit the context of the person to be trained (e.g., planning for the future may have more functionality for young adults than for fourth age pensioners).

A minority of the interventions listed in Table 2 are aimed at raising awareness of how one lives and feels. Techniques used for this purpose are mood tracking, activity diaries, and life reviews. The idea behind these approaches is that such awareness will help you to find a way of life that feels good for you, as explained in more detail in Bakker et al. (2020). The limitations are that (1) awareness of positive and negative moods does not automatically enable people to change their lives in a way that enables them to be happier more often, (2) the effect of greater awareness can backfire on happiness when change is not possible, as it only makes people more keenly aware about their misery, and 3) these interventions report high levels of attrition.

From Table 3 we observe that both of the kinds of interventions discussed above yield positive effects on happiness, and from Table 4 we can see that the resulting changes in happiness following raised awareness are in the range between 1 and 23%, which is higher on average than the effects yielded by the other single-method training techniques listed in Table 4. If raising happiness awareness enables people to change their lives in ways so that they become happier, then this has important implications for all the studies used in the research synthesis. All training, and even all filling out surveys in a control group, will raise awareness of how happy one feels. Raising awareness may partly explain the effectiveness of other life skills training.

The fact that mood awareness is effective highlights that pursuing happiness has two sides to it. The first is the effort to milk as much positive affect out of interaction with the world, for example by increasing gratitude, savoring, or avoiding self-defeating lines of reasoning. Positive psychology often aims to create positive feelings along this line of reasoning. Raising mood awareness starts out with the opposite. You can try to do more of what feels good and avoid what feels bad. After all, our emotions can also be thought of as a signal that we are doing well or bad. Negative affect signals that we should try to change something, while positive affect signals that we are doing fine and that we can explore the world (Frijda, 1986). Pursuing happiness is just a much about listening to the messages of our emotions and moods and changing our behavior and the circumstances of life accordingly as it is about creating more pleasant feelings in our current interaction with the world (Bergsma, 2000, 2020).

#### Similarity With Other Overview Studies on the Effect of PPIs

If we compare our results with earlier work on the effects of PPIs on the wider subjective well-being, we can conclude that our results are in line with these. Sin and Lyubomirsky (2009), Bolier et al. (2013), and White et al. (2019) describe similar modest and positive effects, which suggests that the heterogeneous subjective well-being measures used in these meta-analyses have not changed the average effect.

## Conclusion

Participation in a happiness training course is typically followed by a rise in happiness, in particular participation in training that focuses on multiple mental skills and/or happiness awareness techniques and by people voluntary looking to achieve greater happiness. Hence offering happiness training to employees is a good option for organizations that want to boost their productivity through employee happiness.

## Author Contributions

All authors listed have made a substantial, direct and intellectual contribution to the work, and approved it for publication.

## Conflict of Interest

The authors declare that the research was conducted in the absence of any commercial or financial relationships that could be construed as a potential conflict of interest.

## Footnotes

1 https://worlddatabaseofhappiness.eur.nl/search-the-database/correlational-findings/#id=0zYMIXQB9uCjuYHDZDYa

2 https://worlddatabaseofhappiness.eur.nl/search-the-database/correlational-findings/#id=1DYPIXQB9uCjuYHDZTYe
