# Supplementary material for: Will Happiness-Trainings Make Us Happier? A Research Synthesis Using an Online Findings-Archive
Source: Front Psychol. 2020 Nov 17;11:1953. doi: 10.3389/fpsyg.2020.01953 (PMC7707103; doi:10.3389/fpsyg.2020.01953)
Supplement: Supplementary file 1 [file Data_Sheet_1.docx]

Ad Bergsma, Ivonne Buijt & Ruut Veenhoven (2020)
**Will happiness-trainings make us happier? A research synthesis using an online findings-archive**In: Llewellyn Ellardus Van Zyl & Sebastiaan Rothmann (eds.) Positive Organizational Interventions: Contemporary Theories, Approaches and Applications, in Frontiers in Psychology - section Organizational Psychology. 11:1953. doi: 10.3389/fpsyg.2020.01953

**ONLINE SUPPLEMENT TO TABLES 6-10: Details on numbers of participants**

|  | **METHOD OF INVESTIGATION** | | | | | | |
| --- | --- | --- | --- | --- | --- | --- | --- |
|  | **Cross-sectional** had training vs  had not | **Longitudinal** before vs after training ‒ | | | | | |
|  |  | ***Change in treated group only*** | | ***Difference with change in control group*** | | | |
|  |  | *Post intervention* | *After follow-up* | *Post intervention* | | *After follow-up* | |
| **Participants** | | | | | | | |
| **All studies**  Number of studies | 8 | 39 | 22 | | 31 | | 14 |
| Participants(n) | 630 | 3539 | 2126 | | 3562 | | 1664 |
| Mean n  Median n; range | 79  61; 20-192 | 91  43; 10-606 | 97  53; 10-606 | | 115  73; 23-631 | | 119  89; 37-360 |
|  |  |  |  | |  | |  |
| **Single nature**  Nr of studies  Participants (n)  Mean n  Median n; range | 8  630  79  61; 20-192 | 29  2549  88  43;13-606 | 16  1647  103  74; 20-606 | | 24  2594  108  66; 23-631 | | 11  1146  104  84;40-349 |
|  |  |  |  | |  | |  |
| **Multiple kinds**  Number of studies  Participants (n)  Mean n  Median n; range | 0 | 10  990  99  48;10-306 | 6  479  80  47; 10-306 | | 7  968  138  80; 37-360 | | 3  518  173  121; 37-360 |
| **Online e-training**  Number of studies  Participants (n)  Mean n  Median n; range | 0 | 4  735  184  157; 94-327 | 2  275  138  83-192 | | 1  349 | | 1  349 |
|  |  |  |  | |  | |  |
| **Offline guided training**  Number of studies  Participants (n)  Mean n  Median n; range | 8  630  79  61; 20-192 | 35  2804  80  41; 10-606 | 20  1851  93  48; 10-606 | | 30  3213  107  72; 23-631 | | 13  1315  101  79; 37-360 |
|  |  |  |  | |  | |  |
| **Care setting**  Number of studies  Participants (n)  Mean n  Median n; range | 3  127  42  31; 31-65 | 2  56  28  26-30 | 2  107  54  24-83 | | 2  103  52  44-59 | | 1  152 |
| **Educational setting**  Number of studies  Participants (n)  Mean n  Median n; range | 2  322  161  130-192 | 15  1623  108  41; 14-606 | 7  953  136  44; 20-606 | | 14  2011  144  76; 23-631 | | 4  283  71  73; 40-96 |
| **Work setting**  Number of studies  Participants (n)  Mean n  Median n; range | 0 | 2  64  32  10-54 | 3  163  54  54; 10-99 | | 0 | | 0 |
| **Voluntary participation**  Number of studies  Participants (n)  Mean n  Median n; range | 7  500  71  56; 20-192 | 24  1991  83  57; 10-327 | 15  1245  83  69; 10-306 | | 17  2023  119  84; 26-360 | | 10  1440  144  121; 60-360 |
| Paid of study credit  Number of studies  Participants (n)  Mean n  Median n; range | 0 | 5  122  24  22; 16-33 | 2  49  25  16-33 | | 5  253  51  50; 37-64 | | 2  95  48  37-58 |
| **Mandatory**  Number of studies  Participants (n)  Mean n  Median n; range | 1  130 | 10  1426  143  43; 14-606 | 5  832  166  44; 20-606 | | 9  1286  143  80; 23-631 | | 2  129  65  40-89 |
| **Children**  Number of studies  Participants (n)  Mean n  Median n; range | 1  130 | 4  1051  263  213; 44-606 | 3  670  223  44; 20-606 | | 3  760  253  89; 40-631 | | 2  129  65  40-89 |
| **University students**  Number of studies  Participants (n)  Mean n  Median n; range | 2  297  149  105-192 | 19  1370  72  36; 14-306 | 8  840  105  66; 16-306 | | 20  2251  113  76; 23-360 | | 8  1100  138  88; 37-360 |
| **Elderly**  Number of studies  Participants (n)  Mean n  Median n; range | 0 | 1 | 1 | | 0 | | 0 |
